# A 6-week randomized-controlled field study: effect of isokinetic eccentric resistance training on strength, flexibility and muscle structure of the shoulder external rotators in male junior handball players

**DOI:** 10.3389/fphys.2024.1368033

**Published:** 2024-03-07

**Authors:** Sebastian Vetter, Maren Witt, Pierre Hepp, Axel Schleichardt, Stefan Schleifenbaum, Christian Roth, Timm Denecke, Jeanette Henkelmann, Hans-Peter Köhler

**Affiliations:** ^1^ Department of Biomechanics in Sports, Leipzig University, Leipzig, Germany; ^2^ Department of Orthopaedics, Trauma and Plastic Surgery, Leipzig University, Leipzig, Germany; ^3^ Department of Biomechanics, Institute for Applied Training Science, Leipzig, Germany; ^4^ Department of Pediatric Radiology, Leipzig University, Leipzig, Germany; ^5^ Department of Diagnostic and Interventional Radiology, Leipzig University, Leipzig, Germany

**Keywords:** diffusion MRI, prevention, dynamometer, strength training, range of motion, rotator cuff, hypertrophy, fibres

## Abstract

**Background:** Team handball involves a tremendous amount of shoulder motion with high forces during repeated extended external range of motion. This causes shoulder complaints and overuse injuries. While eccentric training for the lower extremity shows preventive effects by improving strength, range of motion and fascicle length, there is a research gap for the shoulder joint and for advanced tissue characterization using diffusion tensor imaging.

**Objectives:** To investigate the effects of 6-week eccentric isokinetic resistance training on strength, flexibility, and fiber architecture characteristics of the external rotators compared to an active control group in junior male handball players.

**Methods:** 15 subjects were randomly assigned to the eccentric training group and 14 subjects to the active control group (conventional preventive training). Primary outcome measures were eccentric and concentric isokinetic strength of the external rotators, range of motion, and muscle fascicle length and fascicle volume.

**Results:** The intervention group, showed significant changes in eccentric strength (+15%). The supraspinatus and infraspinatus muscles showed significant increases in fascicle length (+13% and +8%), and in fractional anisotropy (+9% and +6%), which were significantly different from the control group.

**Conclusion:** Eccentric isokinetic training has a significant effect on the function and macroscopic structure of the shoulder external rotators in male junior handball players. While strength parameters and muscle structure improved, range of motion did not change. This research helps understanding the physiology of muscle and the role of eccentric training on shoulder function and muscle structure. Furthermore, DTI was found to be a promising tool for advanced tissue characterization, and the *in vivo* derived data can also serve as model input variables and as a possibility to extend existing *ex-vivo* muscle models. Future research is needed for functional and structural changes following convenient eccentric field exercises.

## 1 Introduction

The frequency and severity of shoulder injuries is an ongoing problem in overhead sports as the shoulder is the fourth most commonly injured joint at 9.1% (German Statutory Accident Insurance, VBG). The prevalence of shoulder injuries reaches up to 36%, and in baseball the return to performance rate is dramatically low on 7% (Fedoriw et al., 2014). As an example for early career deficits in shoulder, 69% of junior javelin throwers revealed posterosuperior intraosseous cysts of the humeral head with a size larger than >3 mm in their throwing shoulder compared to 15% in the contralateral shoulder. Even when age cannot be found as a risk factor for injury, most future problems in shoulder can be addressed to chronic overuse beginning in early high performance sports career stage (Astolfi et al., 2015; Achenbach et al., 2020; Achenbach and Luig, 2020; Tooth et al., 2020).

In team handball, the shoulder is frequently subjected to high loads in critical joint positions (Van den Tillaar and Ettema, 2007; Wagner et al., 2010). The standing throw shows the highest kinematic values, in addition to the pivot and jumping throws (which account for 75% of the throws in competition according to Wagner et al., 2008). Elite male handball players can reach up to 130° of external range of motion (ROM) during a standing throw and −65° of external ROM at ball release (Van den Tillaar and Ettema, 2007), as well as a maximum angular velocity of 96 rad/s and a ball velocity of 24 m/s in internal rotation during a standing throw with a run-up (Wagner et al., 2010). These repeated high demands lead to adaptations resulting in a condition known as thrower’s shoulder, characterized by a glenohumeral internal rotation deficit (GIRD) and extended external rotation (Lubiatowski et al., 2018; Achenbach et al., 2020; Asker et al., 2020). The consequences are internal impingement syndrome, tendinopathies and rotator cuff tears (Burkhart et al., 2003; Jost et al., 2005; Doyscher et al., 2014; Lubiatowski et al., 2018). Overuse injuries to the shoulder are common among youth handball players, with 25% suffering each season (Aasheim et al., 2018). Rehabilitation for these injuries typically takes longer than for most joints (Asker et al., 2018).

To prevent throwing injuries to the shoulder there are several options. A typical approach is to improve ROM and muscle strength, which are considered risk factors for injury (Murphy et al., 2003; Hoppe et al., 2022). A GIRD above 20° and an isometric strength ratio of 0.78 is typically found for a throwing shoulder with high risk for injury (Lubiatowski et al., 2018; Achenbach et al., 2020). In handball, there is a gap in prevention studies for the shoulder (Achenbach et al., 2020), while prevention studies are desperately needed because commonly prescribed exercise strategies for the shoulder do not reduce the risk of overuse injuries in handball (Achenbach et al., 2022). To the best of our knowledge, only Andersson et al. (2017) investigated the preventive effects of a strengthening program for the external rotators and found an increase in strength and a 28% decrease in shoulder injuries, providing a little indication of the potential for shoulder prevention exercises.

To increase the impact of training interventions by addressing multiple risk factors at the same time, innovative approaches to strength training for the lower limb use eccentric-focused protocols. Recent review articles show that not only flexibility (O’Sullivan et al., 2012) but also strength (Cuthbert et al., 2020) can be increased more effectively by eccentric-only exercises compared to concentric-focused or passive stretching exercises. A recent meta-analysis of 8,459 athletes showed that incorporating eccentric exercises into athletic training can reduce the risk of injury by 50%. (Van Dyk et al., 2019). This effectiveness in performance enhancement and injury prevention may be explained by high impact changes in muscle structure. Eccentric training produces tissue damage (Franchi et al., 2017) that promotes hypertrophic reactions in terms of increasing muscle cross-sectional-area and muscle volume (Gérard et al., 2020). Besides muscle volume changes, a muscle fascicle lengthening (Franchi et al., 2017) occurs after eccentric strength training compared to concentric training (Vetter et al., 2022) or stretching exercises (Freitas and Mil-Homens, 2015). According to Butterfield et al. (2005), eccentric training causes a rightward shift in the force-length relationship, whereas concentric training would likely have the opposite effect. This would be dysfunctional for a muscle that is regularly subjected to eccentric contractions. Since a shortened muscle fiber has also been suggested as a risk factor for lower extremity injury (Timmins et al., 2016), and resistance training targeting muscle structural changes is also advised for the shoulder (Yagiz et al., 2022), eccentric training may be a beneficial intervention for the shoulder as well.

In the last decade, an increasing number of laboratory eccentric training studies have been published on functional and structural changes for the lower limbs, but not for the shoulder (Vetter et al., 2022). Another research gap exists for the investigation of three-dimensional changes in muscle architecture (Suskens et al., 2022). For this reason, quantitative magnetic resonance imaging (MRI) techniques are increasingly being used for technologically advanced tissue characterization. MRI-based diffusion tensor imaging of muscle (mDTI) is an innovative approach for showing the fiber arrangement for the whole muscle volume (Damon et al., 2011). This technique provides a valid (Schenk et al., 2013), and reliable (Bolsterlee et al., 2019; Forsting et al., 2021; Vetter et al., 2023b) calculation of muscle fiber metrics and may solve problems existing in the use of 2D ultrasound imaging (Yagiz et al., 2021; Charles et al., 2022).

The aim of this study was to investigate the structural and functional changes of the dominant throwing shoulder in competitive male junior handball players after 6 weeks of isokinetic eccentric strength training compared to an active control group. The study used isokinetic dynamometry and mDTI to assess changes in the external rotators’ ROM, eccentric and concentric strength, muscle fascicle length (FL), absolute fascicle volume, and fractional anisotropy (FA). Additionally, the study evaluated strength and flexibility parameters for the internal rotators.

## 2 Materials and methods

This study is a randomized-controlled trial with an exercise-based intervention for the external rotators of the throwing shoulder. Five days before (pre) and after (post) the training phase, functional and structural parameters were examined for the dominant throwing shoulder using mDTI and an isokinetic dynamometer as described below. Participants were recruited locally during the final preparations and the beginning of the handball season of the German national junior league in late August 2022. The study can be found in the German Register for Clinical Trials (DRKS00028885). The study protocol was approved by the Leipzig University ethics committee (ethical approval number: 362/21-ek). The study was conducted in accordance with the relevant guidelines and regulations. Written informed consent was obtained from all subjects.

### 2.1 Participants

The sample consisted of 29 male junior handball players (German male handball division A and U23) from a local elite handball club (18.0 ± 1.6 years; 186.8 ± 6.3 cm; 84.8 ± 11.3 kg). Similar levels of physical activity (∼3 h per day) were found between the handball players, who were closely coached by the same athletics trainer. Sample size was based on a preliminary study (Vetter et al., 2023a) and on comparable studies that calculated or suggested at least 13 participants per group (Kim et al., 2015; Marušič et al., 2020). The participants were randomly assigned to one of the two groups according to their playing position. Both groups included all playing positions and players with left and right throwing hands. For eligibility, participants had to show a healthy throwing shoulder. Exclusion criteria were regular medication that may affect the adaptability, muscle-nerve diseases, discomfort, pain or known lesions of the right shoulder. Further, subjects were told to stop with unusual physical activity or intense training 5 days prior to data collection.

### 2.2 Intervention group eccentric exercise

12 eccentric isokinetic strength training sessions for the external rotators were performed within 6 weeks (twice weekly). The training was performed on a BTE Primus RS isokinetic dynamometer (Baltimore Therapeutic Equipment Company, Hanover, MD, United States of America). All subjects completed two familiarization sessions prior to the intervention phase. The eccentric isokinetic training was incorporated into the regular athletic training routine, but replaced specific preventive shoulder exercises performed instead by the active control group. To generate eccentric force through the external rotators, the subject was instructed to pull the lever arm maximally in the external direction while the isokinetic machine pulled the lever arm in the direction of internal rotation. The eccentric exercise protocol was standardized as follows: 5 × 10 repetitions (maximum intensity) with 90 s rest between repetitions, 120° total training ROM (60° internal and external rotation), 30°/s movement speed, supine position with 90° elbow flexion and neutral hand position, 48–72 h rest between training sessions. After 3 weeks of intervention, the training ROM was shifted 15° toward the internal ROM and the load magnitude was increased based on intensity of the previous three sessions. This training regime was illustrated and assessed by a preliminary investigation (Vetter et al., 2023a).

### 2.3 Control group conventional training

The control group performed free-weight rotator cuff exercises, such as shoulder abduction, front rowing, or shoulder rotation, with a non-specific focus on the concentric or eccentric phase. This training regime is based on the recommendations by the local handball club and the German Handball Federation. The training load was adjusted to match that of the eccentric training group in terms of weeks, days of training, sets, and repetitions in each session.

### 2.4 Isokinetic testing

Flexibility and strength tests were performed prior to MRI on the IsoMed 2000 isokinetic dynamometer (D&R Ferstl GmbH, Hemau, Germany). As described for eccentric training, subjects were tested for the dominant throwing shoulder in the supine position with fixed shoulder-arm-hand joints (90° shoulder abduction, 90° elbow flexion). Straps and adapters with pads were used on the isokinetic device to prevent whole body and shoulder elevation. For information on the experimental setup, please refer to Vetter et al. (2023a). In terms of order, a 10-min warm-up on the rowing machine was performed first. Then, the isokinetic diagnostics began with active and passive ROM tests, followed by strength tests at 60°/s and then at 30°/s isokinetic velocity, always separated for concentric and eccentric movement. About 1 min of rest was allowed between tests. For the flexibility and strength diagnostics, the external and internal ROMs were determined based on the neutral position (0°) on the isokinetic device, which was defined when the lever arm was perpendicular (90°) to the body.

The active ROM test consisted of two familiarization repetitions and five consecutive test trials that dynamically alternated between internal and external rotation direction. The participant was instructed to perform a slow, active concentric muscle contraction to produce a stretch in the antagonistic muscle group. The maximum active ROM was measured when the participant was no longer able to continue producing a stretch with gentle and controlled movements. Passive ROM-tests were performed at an isokinetic speed of 10°/s with eyes closed. The passive stretch consisted of ten trials over the total ROM and alternating between the internal and external rotation. Maximum passive ROM was defined when subjects either reached 9 Nm or 100° of internal rotation and 140° of external rotation.

The maximal strength tests were performed separately for concentric and eccentric, also alternating between internal and external rotation. After two familiarization trials, three repetitions were performed over an amplitude of 150° (60° internal rotation and 90° external rotation) at an isokinetic velocity of 60°/s and then 30°/s. All tests were conducted in the same order. The position of each subject, the dynamometer arm and gravity correction as well as the settings were individualized at pre testing and applied in the same way for the post testing. The data were recorded on the isokinetic dynamometer with a recording frequency of 200 Hz.

### 2.5 Diffusion tensor imaging

In supine position MRI scans were performed using a 3-T Siemens MAGNETOM Prisma scanner (Siemens Healthcare, Erlangen, Germany) and a shoulder coil (XL, 16-channel). Commercial T1-weighted (T1w) and diffusion-weighted imaging sequences (DWI) were used. The total scan time was 12 minutes. The volunteers lay in a head first supine position with the right arm in the neutral position and the hand supinated. For the T1w the following settings were acquired: TR/TE = 492/20 m, slice thickness = 0.7 mm, flip angle = 120°, FOV = 180 × 180 mm^2^, matrix = 256 × 256 mm^2^. The following 2D echo planar DWI sequence was acquired as follows: TR/TE = 6100/69 m, slice thickness = 4 mm, flip angle = 90°, FOV = 240 × 240 mm^2^, matrix = 122 × 122 mm^2^, 48 diffusion sampling directions with b = 400 s/mm^2^.

### 2.6 Data processing

The isokinetic dynamometer data were processed using MATLAB v. R2022a (MathWorks, Natick, United States of America). Raw-data were filtered using a fifth order, zero-lag Butterworth low-pass filter at a cut-off frequency of 6 Hz. Afterwards, the torque and angle data were cut according to repetition number and movement direction (internal or external rotation). The acceleration and deceleration phases were excluded from each repetition, retaining only the interval with the desired isokinetic velocity. The maximum and mean torque during the isokinetic phase were then determined. For further analysis, the torque and angle data were normalized to body mass (Forthomme et al., 2011; Zanca et al., 2011). In order to compare torque-angle-curves, the data were interpolated to 101 samples for one-dimensional Statistical Parametric Mapping (SPM), (Pataky, 2011). Flexibility analysis were based on the average of five trials of each ROM test. For these trials, a three-parametric e-function was fitted (Vetter et al., 2023a). This enabled analyses for submaximal ROM and maximal ROM, and changes in the passive torque-angle curves using SPM analysis.

MRI data were processed using Mimics Materialise v. 24.0 (Leuven, Belgium) for prior muscle segmentation and DSI Studio (v. 3 December 2021, http://dsi-studio.labsolver.org) for muscle tractography. The T1w images from the MRI were used for manual segmentation of the supraspinatus and infraspinatus muscles. After extraction of the segmented muscle volume of interest, the DWI were corrected for motion and eddy current distortion using DSI Studio’s integrated FSL eddy current correction. The DWI were visually inspected by two independent raters. If artifacts or field of view did not allow full muscle analysis, subjects were excluded. Tractography was performed using specific stopping criteria defined in the previous work (Vetter et al., 2023b). The deterministic fiber tracking is based on an Runge-Kutta fourth-order algorithm. After tractography, DSI Studio was used to calculate the muscle FL and the muscle fascicle volume using the integrated statistics tool. This method has been previously evaluated (Vetter et al., 2023b).

### 2.7 Statistical analysis

Statistics and graphs were performed and built using SPSS v. 27 (IBM, Armonk, New York, United States of America) and MATLAB v. R2022a (MathWorks, Natick, United States of America). Descriptive results were based on mean values and the standard deviation (±). Participants were excluded from further analysis if the z-transformed values reached 2.5. Repeated measures mixed multivariate analysis of variance (MANOVA) were used to show overall differences. The factors were group (Intervention and control), time (pre and post), mode (eccentric and concentric), and speed (30°/s and 60°/s). Post-hoc analysis was performed using repeated measures univariate analysis of variance (ANOVA) and t-tests for within group pre-post comparisons. The level of significance was set to α = 0.05. Further, to compare the morphology of the angle-torque and passive torque-angle trajectories, the interpolated curves were compared using SPM analysis. For this purpose, an ANOVA with repeated measures was chosen.

## 3 Results

Statistics were performed on 14 subjects from the intervention group. These subjects had a mean age of 17.9 ± 1.1 years, a height of 188.6 ± 6.8 cm, and a body weight of 89.2 ± 11.6 kg. The group consisted of six Junior A and eight U23 players, with nine right-handed and five left-handed players. There were four wings, six backcourt, and three pivot players, as well as one goalkeeper. The study’s control group comprised 12 subjects (17.4 ± 1.2 years, 183.8 ± 5.2 cm, 77.9 ± 8.5 kg, seven Junior A and five U23 players, nine right-handed and three left-handed players, seven wings, three backcourt, one pivot player, and one goalkeeper). These subjects completed 12 training sessions within 6 weeks with sufficient compliance. However, from this sample, 22 participants (13 intervention group) were eligible for supraspinatus fascicle analyses due to limited field of view. 25 participants (14 intervention group) were included in the ROM analyses, as four participants were not available for functional testing due to shoulder problems or transfer to another handball club.

### 3.1 Primary outcome measures

Functional and structural changes for the trained dominant throwing shoulder external rotator cuff muscles were analyzed for pre-post changes within the intervention and control group and for differences in changes between the two groups as shown in Table 1.

**TABLE 1:** Primary outcome measures *post hoc* comparisons.

Parameter		Intervention					Control					Dif
		Pre	Post	∆%	p	d	Pre	Post	∆%	p	d	p
Eccentric strength (*N*m)												
Mean torque	30°/s	30.79 ± 7.66	34.38 ± 9.68	11.66	.053	.465	25.95 ± 5.72	26.27 ± 6.65	1.27	.777	.101	.121
	60°/s	34.91 ± 6.85	38.02 ± 7.48	8.91	.022	.593	28.12 ± 5.29	30.27 ± 8.29	7.65	.293	.334	.330
Peak torque	30°/s	39.56 ± 9.32	45.63 ± 11.13	15.34	.011	.692	35.63 ± 6.28	35.93 ± 6.10	0.84	.777	.088	.035
	60°/s	44.06 ± 7.64	49.31 ± 6.77	11.92	.002	.950	37.03 ± 7.24	40.57 ± 7.67	9.56	.062	.632	.253
Concentric strength (*N*m)												
Mean torque	30°/s	23.24 ± 4.56	24.47 ± 6.49	5.29	.152	.286	20.10 ± 4.55	20.81 ± 4.77	3.53	.341	.302	.394
	60°/s	26.48 ± 5.38	27.97 ± 6.04	5.63	.064	.435	22.52 ± 4.16	23.25 ± 4.80	3.24	.367	.285	.299
Peak torque	30°/s	30.98 ± 5.98	33.31 ± 7.51	7.52	.077	.404	27.38 ± 4.40	27.55 ± 5.37	0.62	.835	.064	.145
	60°/s	34.09 ± 6.94	36.48 ± 6.45	7.01	.032	.539	29.33 ± 4.95	29.44 ± 5.54	0.38	.931	.027	.110
Flexibility (internal rotation)												
Active	ROM (°)	69.88 ± 6.85	70.35 ± 10.93	0.67	.410	.062	72.46 ± 14.11	72.68 ± 12.93	0.30	.451	.038	.464
Passive	ROMsub (°)	55.91 ± 12.84	52.59 ± 13.30	−5.94	.060	.444	59.46 ± 13.96	58.27 ± 13.44	−2.00	.590	.168	.239
	ROM (°)	79.55 ± 10.03	77.54 ± 11.27	−2.53	.179	.255	82.00 ± 9.09	83.25 ± 11.25	1.52	.356	.292	.116

Columns indicate the absolute pre and post-test values and standard deviation (±) for each group, percentage change (∆%) and significant pre-post differences within groups (p), cohens d effect size (d), and in column “Dif” the statistical significance for between-group differences (normalized to body mass for strength). Nm, newton meter; ROMsub, submaximal range of motion; °, angle degree; °/s, degrees per second (movement velocity).

For strength measures (normalized to body mass), an overall four-way mixed measures MANOVA for group x time x mode x speed showed a main effect for the factor time (Wilks-Lambda = .699; *F* (2,22) = 4.74; *p* = .019; ηp^2^ = . 301), an interaction effect for time x mode (Wilks-Lambda = .699; *F* (2,22) = 4.74; *p* = .019; ηp^2^ = .301) and an interaction effect for the factors time x speed (Wilks-Lambda = .691, *F* (2,22) = 4.92; *p* = .017; ηp^2^ = .309) but not for time x group (Wilks-Lambda = .832; *F* (2,22) = 4.74; *p* = .132; ηp^2^ = .168). Post-hoc analysis revealed a group × time interaction in peak torque and thus a training effect for the trained 30°/s eccentric strength (*F* (1,23) = 3.628, *p* = .035; ηp^2^ = .136). The main effect time showed group independent significant differences from pre to post (*F* (1,23) = 4.82; *p* = .038; ηp^2^ = .173).

For flexibility measures, a two-way MANOVA (group x time) revealed no interaction effect for the passive ROM test for the external rotators (Wilks-Lambda = .945; *F* (2,22) = <1; *p* = .054; ηp^2^ = .055). Furthermore, the active ROM revealed no significant interaction effect for the factors group x time (*F* (1,23) = <1; *p* = .773; ηp^2^ = .004). Further *post hoc* comparisons for strength and flexibility parameters can be found in Table 1.

For analysis of the supraspinatus muscle fascicle, a two-way MANOVA showed a group x time-interaction effect (Wilks-Lambda = .492; *F* (3,17) = 5.86; *p* = .006; ηp^2^ = .508). Furthermore, ANOVA showed a significant change in fascicle length (*F* (1,19) = 3.37; *p* = .041; ηp^2^ = .151) and FA (*F* (1,19) = 3.92; *p* = .062; ηp^2^ = .171). The infraspinatus muscle also showed positive interaction effects for group x time (Wilks-Lambda = .281; *F* (3,11) = 9.38; *p* = .002; ηp^2^ = .719). The univariate analysis revealed a positive change for fascicle length (*F* (1,13) = 4.17; *p* = .031; ηp^2^ = .243) and FA (*F* (1,13) = 10.25; *p* = .007; ηp^2^ = .441). Further *post hoc* comparisons can be found in Table 2.

**TABLE 2:** Post-hoc results for muscle structural measures.

Muscle	Parameter	Intervention					Control					Dif
		Pre	Post	∆%	p	d	Pre	Post	∆%	p	d	p
Infraspinatus	Fascicle length (mm)	69.47 ± 13.31	75.23 ± 7.82	8.29	.038	.632	81.04 ± 7.98	77.35 ± 6.82	−4.55	.287	.549	.031
	Fascicle volume (mm^3^)	273.78 ± 79.84	291.49 ± 48.58	6.47	.248	.224	305.05 ± 61.38	297.60 ± 19.73	−2.44	.749	.154	.265
	Fractional Anisotropy	.251 ± .027	.273 ± .024	8.85	.002	1.251	.279 ± .020	.267 ± .014	−4.33	.307	.523	.004
Supraspinatus	Fascicle length (mm)	37.47 ± 6.42	42.38 ± 6.17	13.10	.017	.663	39.99 ± 8.00	39.59 ± 6.45	−1.00	.819	.079	.041
	Fascicle volume (mm^3^)	153.18 ± 37.29	165.65 ± 26.52	8.14	.158	.303	146.65 ± 41.34	164.07 ± 22.37	11.88	.246	.418	.395
	Fractional Anisotropy	.285 ± .027	.303 ± .014	6.32	.028	.584	.295 ± .027	.289 ± .028	−1.90	.386	.306	.031

Columns indicate the absolute pre and post-test values and standard deviation (±) for each group, percentage change (∆%) and significant pre-post differences within groups (p), cohens d effect size (d), and in column “Dif” the statistical significance for between-group differences.

**FIGURE 1:**
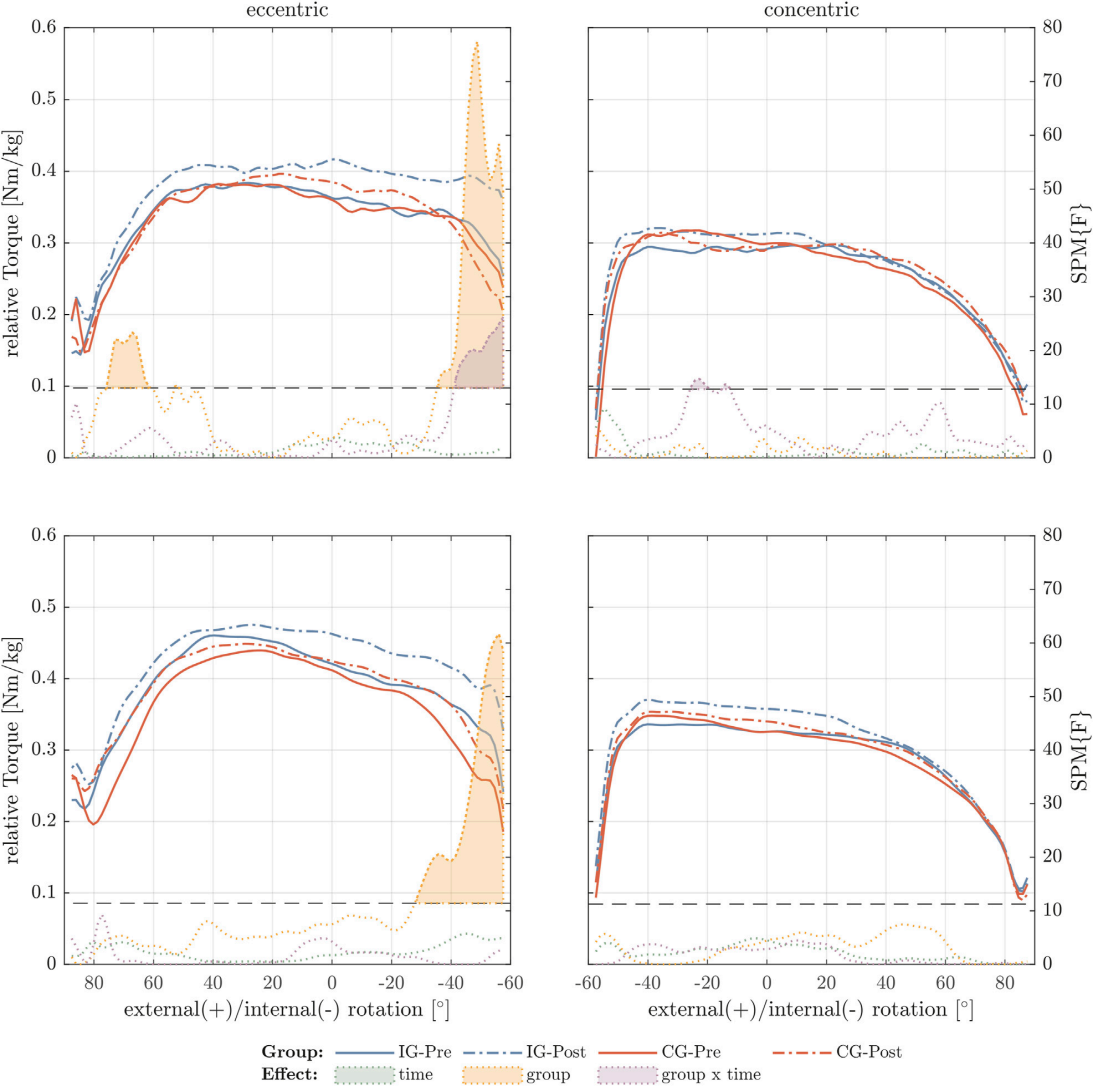
External rotator torque-angle curves under maximal contractive conditions. The figure shows the relative torque-angle curves (Nm/body mass) for 30°/s (top row) and 60°/s (bottom row) for the intervention group (IG) and control group (CG) of pre- and post-test. In addition, *F*-values were calculated for the factors time, group, and time × group using statistical parametric mapping (SPM). Values exceeding the critical *F*-value (dashed horizontal line) show significant differences for the different effects and are filled with the corresponding color.

**FIGURE 2:**
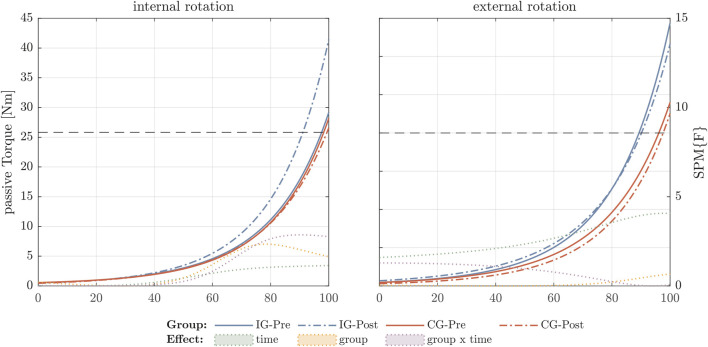
External rotator torque-angle curves under passive condition. The figure shows the absolute torque-angle curves for the intervention group (IG) and control group (CG). The *y*-axis indicates absolute passive torque values during stretching and the *x*-axis the range of motion until maximal stretch (100%). In the *y*-axis on the right side the *F*-values were calculated for the factors time, group, and time × group using statistical parametric mapping (SPM). Values exceeding the critical *F*-value (dashed horizontal line) show significant differences for the different effects and are filled with the corresponding color.

For the external rotator muscles eccentric strength, SPM analysis showed a significant group x time difference and an increase in the torque-angle relationship in between 30° and 60° internal ROM (Figure 1). The passive torque-angle curves did not change statistically significantly, but an increase in passive stiffness for internal rotation in the intervention group can be visually inspected (Figure 2).

### 3.2 Secondary outcome measures

Functional changes for the untrained shoulder internal rotators were analyzed for pre-post changes within the intervention and control groups and for differences in changes between the two groups, as shown in Table 3. Overall, for strength analysis the MANOVA did not reveal any effects for the internal rotator muscles. However, *post hoc* analysis for the intervention group showed that eccentric and concentric strength increased in the 60°/s peak torque (Table 3). For flexibility measures, a two-way MANOVA (group x time) revealed no interaction effect for the passive ROM test for the internal rotator muscles (Wilks-Lambda = .932; *F* (2,22) ≤ 1; *p* = .460; ηp^2^ = .068). In addition, *post hoc* analysis showed positive pre-post changes in passive ROM for the intervention group (Table 3).

**TABLE 3:** Secondary outcome measures *post hoc* comparisons.

Parameter		Intervention					Control					Dif
		Pre	Post	∆%	p	d	Pre	Post	∆%	p	d	p
Eccentric strength (*N*m)												
Mean torque	30°/s	37.46 ± 5.94	38.91 ± 9.07	3.88	.278	.302	31.60 ± 6.39	31.20 ± 7.32	1.27	.735	.105	.259
	60°/s	46.41 ± 6.97	48.62 ± 12.68	4.76	.401	.232	37.79 ± 5.49	39.41 ± 9.72	4.29	.523	.200	.422
Peak torque	30°/s	54.50 ± 9.58	61.52 ± 19.09	12.88	.113	.454	45.76 ± 11.43	46.36 ± 10.34	1.31	.693	.122	.113
	60°/s	61.51 ± 8.61	67.76 ± 18.49	10.16	.153	.405	52.81 ± 9.47	54.49 ± 13.62	3.18	.498	.212	.200
Concentric strength (*N*m)												
Mean torque	30°/s	31.79 ± 6.78	32.52 ± 9.91	2.30	.720	.098	27.27 ± 7.26	26.69 ± 6.87	2.13	.793	.081	.331
	60°/s	35.04 ± 6.07	37.12 ± 8.41	5.94	.100	.474	29.92 ± 5.51	31.51 ± 8.09	5.31	.328	.310	.440
Peak torque	30°/s	46.28 ± 9.02	48.37 ± 14.24	4.52	.473	.198	39.37 ± 9.15	38.35 ± 8.91	−2.60	.713	.114	.222
	60°/s	47.52 ± 7.36	52.37 ± 12.00	10.21	.019	.718	40.44 ± 7.77	42.23 ± 9.43	4.43	.284	.341	.146
Flexibility (external rotation)												
Active	ROM (°)	104.83 ± 7.48	107.88 ± 9.98	2.91	.065	.433	103.92 ± 7.57	108.04 ± 10.08	3.97	.251	.367	.387
Passive	ROMsub (°)	78.55 ± 6.80	81.62 ± 8.76	3.91	.023	.591	80.12 ± 9.01	84.64 ± 8.55	5.64	.088	.570	.294
	ROM (°)	105.54 ± 6.96	108.64 ± 9.11	2.94	.024	.583	108.32 ± 9.07	112.23 ± 9.06	3.61	.125	.504	.379

Columns indicate the absolute pre and post-test values and standard deviation (±) for each group, percentage change (∆%) and significant pre-post differences within groups (p), cohens d effect size (d), and in column “Dif” the statistical significance for between-group differences (normalized to body mass for strength). Nm, N m; ROMsub, submaximal range of motion; °, angle degree; °/s, degrees per second (movement velocity).

**FIGURE 3:**
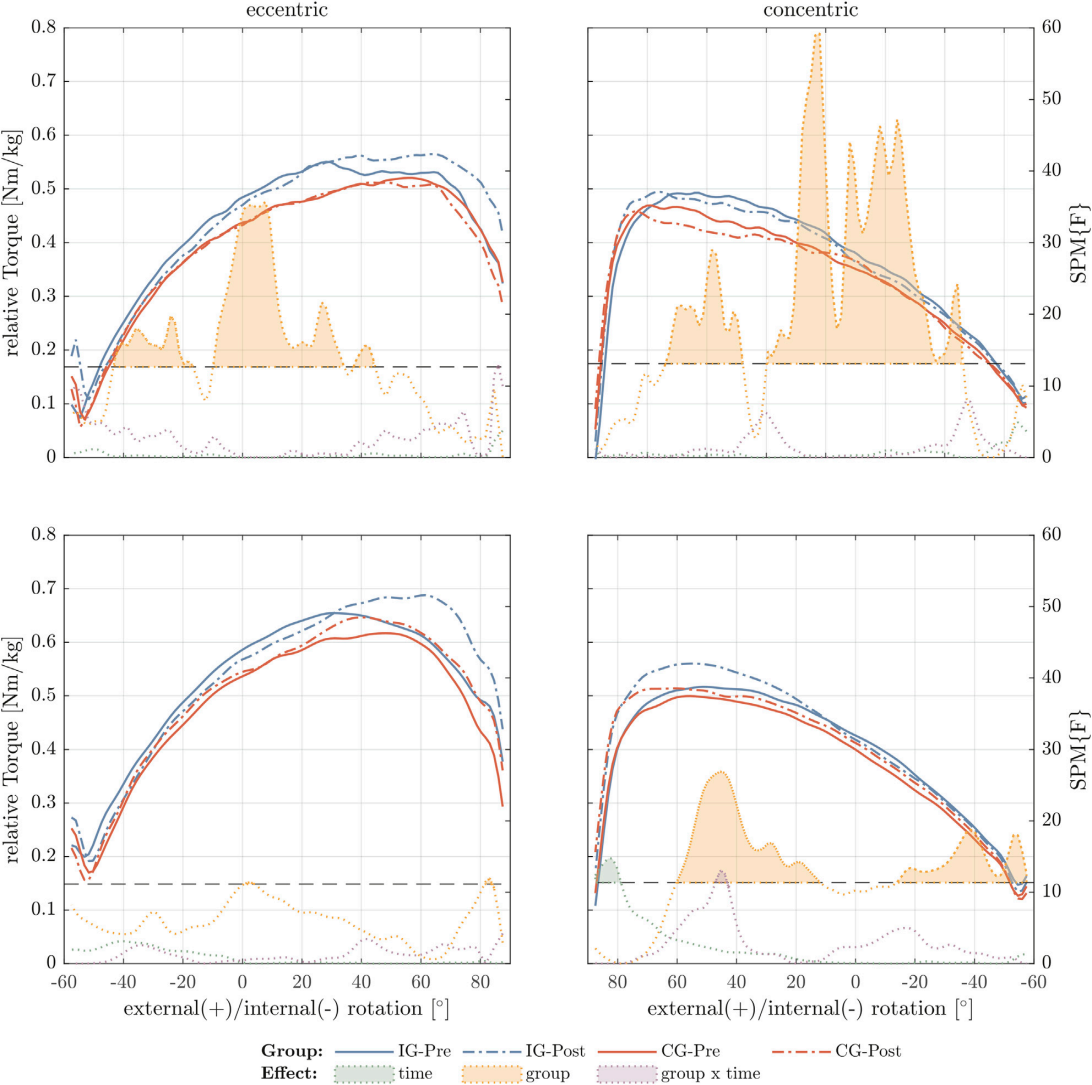
Internal rotator torque-angle curves under maximal contractive conditions. The figure shows the relative torque-angle curves (Nm/body mass) for 30°/s (top row) and 60°/s (bottom row) for the intervention group (IG) and control group (CG) of pre- and post-test. In addition, *F*-values were calculated for the factors time, group, and time × group using statistical parametric mapping (SPM). Values exceeding the critical F-value (dashed horizontal line) show significant differences for the different effects and are filled with the corresponding color.

The internal rotator muscles showed group but not time differences in eccentric and concentric torque-angle relationship using SPM analysis. Differences were found especially in the mid part of ROM and especially for the concentric condition (Figure 3).

## 4 Discussion

The aim of the study was to investigate functional and structural changes in the throwing shoulder of junior male handball players following a 6 week eccentric exercise compared to an active control group. We hypothesized that 6 weeks of 30°/s isokinetic eccentric resistance training for the external rotators would have positive effects on eccentric strength, flexibility, and supraspinatus and infraspinatus muscle FL and muscle fascicle volume. Therefore, *p*-values for *post hoc* comparisons were reported hypothesis dependent. After the eccentric training intervention, the absolute values for eccentric strength increased independent of the movement speed by up to +15%. The supraspinatus fascicle showed +13% lengthening, and +9% change in the DTI metric FA which was significant different to the control group. The infraspinatus muscle FL increased for 8% and the FA for 6%. These findings were significantly different to the control group. No statistically significant changes where observed for flexibility measures and for the internal rotator measures.

### 4.1 Interpretation of results

The results presented were comparable to several eccentric-only intervention studies summarized by Vetter and others (Vetter et al., 2022). In this systematic review, a simple pooled analysis found that eccentric training for the lower limbs was more effective than concentric training in changing more outcome parameters simultaneously, such as eccentric (+19%), concentric (+9%), passive ROM (+9%), and FL (+10%). Concentric strength training was found to be similarly effective in altering strength metrics, but did not alter other injury risk factors such as torque-angle relationship, passive ROM, or muscle FL (Vetter et al., 2022). Eccentric training can be interpreted a beneficial form of prevention training due to the rightward shifts in the eccentric torque-angle relationship (Blazevich et al., 2007). This supports the capability of energy absorption and energy transfer to concentric actions (Mörl et al., 2016; Köhler et al., 2022; Holzer et al., 2023; Köhler and Witt, 2023).

If comparing the present results to multidirectional eccentric training studies for the shoulder, findings were partially similar (Kim et al., 2015; Vetter et al., 2023a). While Kim et al. (2015) showed that eccentric training is superior to concentric in terms of maintaining FL, Vetter et al. (2023a) found a training effect for eccentric strength (+24%), a 4% decrease in ROM, increases in FL (+16%) and muscle fascicle volume (+19%), but no changes occurred in the untrained internal rotators. The rightward shift in the eccentric torque-angle relationship observed in this study confirms the findings of Uhl et al. (2017) and Vetter et al. (2023a), who also recruited healthy, non-specific shoulder trained subjects. In contrast to the force-velocity relationship, the present study found higher concentric torque values at 60°/s compared to those obtained at 30°/s (Table 1; Table 3). Besides other possible explanations, this phenomenon can be explained by sequence effects and the data processing. The torque-angle curves were truncated to the isokinetic interval, as explained in the Methods. Since the morphology of the torque-angle curves differed between both tests, this may have affected the mean torque values. While this phenomenon was also observed in the preliminary study (Vetter et al., 2023a), for most of the outcome measures smaller pre-post changes were found as difference between the studies. This can be partially explained by a so-called plafond effect, as these recruited handball players show higher absolute values in all functional and structural parameters. Another reason why these handball players showed slightly lower changes might be the starting handball season during the intervention phase. A handball season alters shoulder flexibility and strength (Fieseler et al., 2015; Asker et al., 2020) which might be explained with chronic overuse and concurring stimuli. In addition, the described in-season effects on shoulder function (Fieseler et al., 2015) may also explain why external ROM increased in both groups in the present study, but was not statistically significant in the active control group.

An unchanged internal ROM following the eccentric training regime and an increase in passive torque-angle curve was also found by Vetter and others (Vetter et al., 2023a). But in contrast to the mentioned preliminary study (Vetter et al., 2023a), the present sample may benefit from these results since an increase in torque-angle relationship in the final phase of eccentric contraction, and an unchanged ROM can be interpreted as a stabilizing and therefore significant preventive effect for the shoulder. This is because Fieseler et al., (2015) revealed that handball players experience a decrease in internal ROM and show deficits during the season. However, caution must be exercised when interpreting the ROM findings, as other shoulder studies commonly use different testing routines and goniometers (Oyama et al., 2011; Asker et al., 2017; Dejaco et al., 2017).

The study utilized mDTI to exhibit alterations in muscle structural metrics after eccentric training. This technology is innovative and has only been used in two studies in this field (Suskens et al., 2022; Vetter et al., 2023a). Compared to the preliminary study by Vetter et al. (2023a), the metric FA increased significantly by 6%, showing differences between the groups. This increase may indicate an improvement in muscle fiber density and muscle health state, as suggested by Biglands et al. (2020) and Zaraiskaya et al. (2006). The FL metric obtained via mDTI ranged from 3.7 to 4.2 cm between subjects, which is comparable to measurements from anatomical studies using conventional 2D ultrasound imaging or cadaver dissections (Ward et al., 2006; Kim et al., 2007). After eccentric training, FL increased significantly by 13%, compared to 16% in the preliminary study (Vetter et al., 2023a). Changes in FL may result from sarcomere lengthening or sarcomerogenesis (Herzog and de Brito Fontana, 2022). However, it is assumed that muscle fiber remodeling occurs after eccentric shoulder external rotator training (Uhl et al., 2017). The results of the present study indicate sarcomerogenesis due to the observed rightward shift in the eccentric torque-angle relationship, along with a non-significant increase in passive torque during the ROM-test and significant lengthening of the FL (Butterfield et al., 2005; Blazevich et al., 2007). Although the present study revealed non-significant increases in the metric muscle fascicle volume in the supraspinatus muscle (+12% control group, +8% intervention group), this contrasts with the preliminary study (Vetter et al., 2023a). This can be partially explained with lower FL lengthening, which can increase muscle volume (Franchi et al., 2017).

### 4.2 Limitations

Several limitations have to be noted. First, the study design and sample size limited the power of the statistics. To the best of our knowledge, a comparable study by Kim and others (Kim et al., 2015) recruited 13 subjects, while studies of the lower extremity have recruited more, suggesting group sizes of up to 16 subjects (Marušič et al., 2020). The study acknowledges recruiting one of the best junior handball athletes in Germany, which adds value to the handball community. However, this limited sample size and generalizability of the findings. Second, since the isokinetic training machine did not allow to define a target torque, the load magnitude was very heterogenous between subjects. Thus, subjects were told to finish their training session until they reached ±10% of an individual pre-defined workload based on the mean torque from the previous three sessions. Furthermore, the training load and compliance seemed to be playing position dependent. Third, four handball players seemed to have difficulties with the eccentric training and testing. These subjects showed very different torque-angle-relationships especially at the beginning and the end of the ROM during the strength tests. These players explained little discomfort in the shoulder when the experiment was finished. A fourth limitation might occur since this study appears to have primary research characteristics but also characteristics of a field experiment due to the implementation of the intervention in the regular athletics training regime. However, the use of an isokinetic dynamometer and MRI raised options of standardizations while in training the main goal was to complete twelve sessions within 6 weeks. Fifth, in terms of training exercise, the performed external rotation eccentric exercise seems to be less considered to force training-induced changes in muscle architecture compared to abduction exercises for the external rotator muscles (Kim et al., 2015). However, the idea was to focus on rotational movements based on the broadly known glenohumeral internal rotation deficit as a risk factor for injury (Clarsen et al., 2014; Johnson et al., 2018). Last, besides the isokinetic submaximal passive flexibility test, which seems incomparable to other studies using goniometry (Vetter et al., 2022), MRI showed limitations. A very heterogenous distribution in muscle volume within the recruited handball team led to incomplete representation of the external rotators using the largest available shoulder coil. This has also been explained in a preliminary study (Vetter et al., 2023a). Following these difficulties, subjects showed different tract density after fiber tracking. Because of these difficulties, one subject had to be excluded due to artifacts in the supraspinatus muscle and only 15 subjects could be included in the analysis for the infraspinatus muscle due to a limited field of view in the MR images. The ability to focus on the entire rotator cuff was also limited due to the use of a shoulder coil. To optimize image quality for DTI, we focused the DWI scans on the external rotators, which were the target of the eccentric training. Therefore, the fiber tracts for the internal rotators were inadequately represented and could not be analyzed.

## 5 Conclusion and perspectives

Eccentric strength training for the shoulder shows significant improvements in muscle strength and muscle architecture, without decreasing the shoulder rotation ability in trained junior male handball players. The strength of the present study is that it provides new insights into exercise physiology due to its multidirectional study design. Furthermore, as the human shoulder joint is known to be a challenge for any imaging technique, mDTI provided for the first time insights into the shoulder tissue and how it adapts following an eccentric training regime. The implications are, first, that eccentric shoulder strength training could contribute not only to regular primary prevention and rehabilitation, but also to the performance development of the shoulder joint. Second, mDTI was not only found to be an innovative and promising tool for advanced tissue characterization, but also the *in vivo* derived data can serve as model input variables and extend existing muscle models and muscle physiological explanatory approaches. However, future research projects should focus on different eccentric exercises and their multidirectional changes on the shoulder.

## Data Availability

The datasets presented in this study can be found in online repositories. The names of the repository/repositories and accession number(s) can be found below: https://doi.org/10.6084/m9.figshare.24588207.v2.
